# Photosensitized and Photothermal Stimulation of Cellular Membranes by Organic Thin Films and Nanoparticles

**DOI:** 10.3389/fbioe.2022.932877

**Published:** 2022-07-07

**Authors:** Paul L. C. Feyen, Bruno F. E. Matarèse, Laura Urbano, Thais F. Abelha, Hassan Rahmoune, Mark Green, Lea A. Dailey, John C. de Mello, Fabio Benfenati

**Affiliations:** ^1^ Center for Synaptic Neuroscience and Technology, Istituto Italiano di Tecnologia, Genoa, Italy; ^2^ DZNE—German Center for Neurodegenerative Diseases, Munich, Germany; ^3^ Department of Chemistry, Imperial College London, South Kensington Campus, London, United Kingdom; ^4^ Department of Physics, University of Cambridge, Cambridge, United Kingdom; ^5^ Department of Heamatology, University of Cambridge, Cambridge, United Kingdom; ^6^ King’s College London, Institute of Pharmaceutical Science, London, United Kingdom; ^7^ Department of Clinical Pharmaceutical and Biological Sciences, University of Hertfordshire, Hatfield, United Kingdom; ^8^ Laboratory of Optics and Photonics, Institute of Physics, Federal University of Mato Grosso do Sul, Campo Grande, Brazil; ^9^ Department of Chemical Engineering and Biotechnology, University of Cambridge, Cambridge, United Kingdom; ^10^ Department of Physics, King’s College London, London, United Kingdom; ^11^ Department of Pharmaceutical Technology and Biopharmacy, University of Vienna, Vienna, Austria; ^12^ Department of Chemistry, Centre for Organic Electronic Materials, Norwegian University of Science and Technology (NTNU), Trondheim, Norway; ^13^ IRCCS Ospedale Policlinico San Martino, Genoa, Italy

**Keywords:** photothermal, photosensitizer, bioelectronics, solid state–aqueous interphase, reactive oxygen species, conjugated polymers, thin-film, nanoparticle

## Abstract

Conjugated polymers are increasingly exploited for biomedical applications. In this work, we explored the optical characteristics of conjugated polymers of variable chemical structures at multiple levels relevant to biological interfacing, from fluorescence yield to their influence on cellular membrane potential. We systematically compared the performance of conjugated polymer as cast thin films and as nanoparticles stabilized with amphiphilic polyethylene glycol-poly lactic acid-co-glycolic acid (PEG-PLGA). We assessed in both the dark and under illumination the stability of key optoelectronic properties in various environments, including air and biologically relevant physiological saline solutions. We found that photoreduction of oxygen correlates with nanoparticle and film degradation in physiologically relevant media. Using patch-clamp recordings in cell lines and primary neurons, we identified two broad classes of membrane potential response, which correspond to photosensitizer- and photothermal-mediated effects. Last, we introduced a metric named OED_50_ (optical energy for 50% depolarization), which conveys the phototoxic potency of a given agent and thereby its operational photo-safety profile.

## Introduction

Organic semiconductors (OSCs) have emerged as a versatile class of materials with a wide range of applications in biophotonics as photoemitters and phototransducers. They are employed in two ways: 1) encapsulated in solid-state devices that avoid direct contact with the biological environment and 2) open to the environment, with the semiconductor material being in direct contact with the biological medium. Examples of the former approach include organic light–emitting diodes (OLEDs) for optogenetic stimulation (Steude et al., 2015; Matarèse et al., 2019; Morton et al., 2019), while the latter approach, which is the focus of this study, uses predominantly passive devices. As phototransducers, OSCs in contact with aqueous systems/biological tissue have been studied for photothermal cellular stimulation (Martino et al., 2015; Feyen et al., 2016), photosensitized cellular signaling (Abdel Aziz et al., 2020), and as photosensitizer interphases for optically targeted cell death/ablation (Li et al., 2020). As photoemitters, their most widespread application is in combination with optical imaging microscopy for anatomical and sub-cellular structural studies in medicine and biological research, including single-molecule imaging (Jin et al., 2018). Many of the listed applications exploit nanoparticles based on organic conjugated semiconductors (CNPs, conjugated nanoparticles), which have attracted increasing attention for their desirable properties including their nanoscale size, good photostability, high fluorescence efficiency in the visible or near-infrared region (NIR) regions of the electromagnetic spectrum, biocompatibility, and absence of toxic heavy metal ions (Abelha et al., 2020).

The light-induced applications of OSCs described above exploit the relaxation processes following photoexcitation, such as photoluminescence, heat generation due to the internal conversion of the excited state, or electron transfer to a nearby molecule, leading to the generation of radical chemical species. The relaxation processes are coupled and in dynamic competition, which can lead to non-linear responses to light exposure (Peters et al., 2016). On the biological side, effects of undesired relaxation pathways must be carefully considered to pair OSCs effectively and reliably to their intended applications. Due to the complexity of the biochemical systems in which OSCs operate, and the absence of 100% efficiency for any relaxation process, it is important to investigate the multiple effects that OSCs exert on their target system. For example, it is usually undesirable that photoemitter platforms intended for live-cell imaging act as efficient oxygen photosensitizers, as the resultant photogenerated radical species may impair cellular viability. Conversely, it is critical to assess the effect of the biological environment on the OSCs, to ensure a suitable OSC is chosen for a given clinical, diagnostic, or research application (e.g., resistance to sterilization methods and stability in an aqueous system).

In this work, we investigated the photophysical properties of several OSCs and evaluated their biological effects on the cell membrane to determine their suitability for bioelectronic applications such as live-cellular imaging, photo-stimulation, and photo-ablation. In particular, we studied conjugated polymers of varied chemical structures, based on polyfluorene (PF) and polyphenylene vinylene (PPV) in both solid thin-film form and as nanoparticle dispersions, stabilized by polyethylene glycol-poly lactic acid-co-glycolic acid (PEG-PLGA). We assessed the photostability of the conjugated polymers in these two forms under various environmental conditions, ranging from air to biologically relevant cell culture media, and over a wide range of irradiation times. Patch-clamp recordings of human embryonic kidney cell lines (HEK293T) and primary hippocampal neurons revealed two distinct membrane voltage responses to light stimulation: (i) reversible multiphasic photothermal responses and (ii) irreversible depolarization driven by photosensitization of molecular oxygen. The observed responses of cellular membranes to varied material classes and as a function of irradiation power and duration highlight the importance of broadly characterizing and evaluating optical probes such as conjugated polymers for biological applications.

## Materials and Methods

### Material Toolkit Selection

Phenyl alkoxyphenyl PPV copolymer conjugated polymers Livilux™ PDO-124 Super Orange (SO-PPV) and Livilux™ PDY-132 Super Yellow (SY-PPV) were supplied by Merck, Germany. Poly (9, 9-di-n-octyl-2,7-fluorene) (PFO), poly (9,9-dioctylfluorene-2,1,3-benzothiadiazole) (F8BT), poly (2-methoxy-5 -(2′-ethylhexyloxy)-1,4-phenylene vinylene) (MEH-PPV), poly (2.5-di (hexyloxy)cyanotere-phthalylidene) (CN-PPV), and the diblock copolymer poly (ethylene glycol) methyl ether-block-poly (lactide-co-glycolide) with 50:50 ratio of lactide/glycolide (PEG_5K_-PLGA_55K_), and tetrahydrofuran (THF ReagentPlus^®^, ≥99.0%, catalog # 178810)) were supplied by Sigma-Aldrich Corporation (St. Louis, MO, United States).

### Thin-Film and Nanoparticle Preparation.

Solid-state thin films were prepared from 5 mg/ml solutions, using a spin speed of 1,200 rpm. Conjugated polymer nanoparticles were prepared by the solvent displacement technique, with a weight ratio of 1:10 (conjugated polymer:PEG_5K_-PLGA_55K_) (Abelha et al., 2019). A total of 4 ml of THF solution (0.9 mg/ml polymers) was added dropwise to 20 ml of water at room temperature, and the mixture was stirred until THF had completely evaporated. Formulations containing 100% PEG_5K_-PLGA_55K_ and 100% CN-PPV were also prepared with the same polymer concentration. The resulting solutions had a total solid content of 0.2 mg/ml, which were concentrated to a minimum of 1.0 mg/ml using Amicon®Ultra-15 100K centrifugal filter devices (Merck Millipore, Tullagreen, Ireland).

### Hydrodynamic Diameter and Zeta Potential Characterization.

Hydrodynamic diameters were assessed by dynamic light scattering (DLS) using a Zetasizer NanoZS (Malvern Instruments Ltd., United Kingdom) at 25°C with a scattering angle of 173° and 50 μg/ml final polymer concentration. The zeta potential at 25°C, after sample dilution in 10 mM NaCl to a final polymer concentration of 20 μg/ml, was measured using a Zetasizer NanoZS (Malvern Instruments Ltd., United Kingdom). The product yield and spectroscopic characterization were performed according to previously described methods for similar nanoparticles (Abelha et al., 2017).

### Photophysical Measurements

The UV-vis absorption spectrum was measured using a Shimadzu UV-3600 Plus UV-VIS-NIR spectrophotometer. The samples were measured in a transmission mode using a photomultiplier tube (PMT) detector for the ultraviolet and visible regions, with a blank glass substrate as a reference. Absorption spectra of solid-state thin-film conjugated polymers in THF and organic semiconductor nanoparticles (CNPs) in distilled water were measured using a Shimadzu UV-3101 NIR-Vis-NUV spectrophotometer. Emission spectra of solid-state thin-film conjugated polymers in THF and CNPs in distilled water were measured using a FLS980 photoluminescence spectrophotometer with a xenon arc lamp that covers a range of 230–1,000 nm. The samples were excited at their absorbance peak values and signals were captured using a single-photon–counting PMT detector. Photoluminescence Quantum Yield (PLQY) measurements of solid-state thin-film and CNPs in distilled water were performed in an integrating sphere (De Mello et al., 1997) under N_2_ flow using an Andor Shamrock spectrometer and Andor iDus DU420A-BVF Si detector. Time-resolved PL lifetime measurements of solid-state thin films and CNPs in distilled water were performed with a Time-Correlated Single Photon Counting (TCSPC) module SPC830 (Becker & Hickl, Germany). The module was synchronized to a Ti-Sapphire–pulsed laser source (680–1,080 nm, 80 MHz, 140 fs, Chameleon Vision II, Coherent Inc., Germany). TCSPC data were analyzed by fitting to a two-exponential decay.

### Cell Cultures

The human embryo kidney (HEK293) cells (from ATCC) and human epithelial adenocarcinoma (Hela) cell lines were cultured in Dulbecco’s modified Eagle Medium (DMEM) supplemented with 2 mM l-glutamine. The SV40 immortalized human microglial cells were cultured at 37°C/5% CO_2_ in RPMI-1640 with sodium bicarbonate (Sigma-Aldrich). All cell culture media were supplemented with 10% heat-inactivated fetal bovine serum (FBS; Life Technologies), 2 mM l-glutamine, 100 μg/ml penicillin, and 100 μg/ml streptomycin (Life Technologies). The cells were initially sub-cultured in polystyrene flasks and were detached using 0.25% trypsin (2.5 g/L; Sigma). Primary cultures of hippocampal neurons were prepared from embryonic 18-day rat embryos (Charles River). Briefly, hippocampi were dissociated by a 15-min incubation with 0.25% trypsin at 37°C and cells were plated on poly-l-lysine-coated substrates (0.1% PLL in the Borax solution overnight) in a neurobasal medium supplemented with 2 mM l-glutamine, 2% B27, 100 μg/ml penicillin and 100 μg/ml streptomycin, and with a further 10% horse serum (Life Technologies) in the first 4 h of plating. Glass coverslips were thermally sterilized at 120°C prior to overnight incubation in 0.1% PLL solution prior to the transfer of HEK293, HeLa, and SV40 cell lines. The cultures were maintained at 37°C in a humidified atmosphere containing 5% CO_2_.

### Reactive Oxygen Species (ROS) Analysis.

ROS generation by HeLa, HEK, and SV40 cell lines was measured by confocal microscopy and a fluorescence TECAN Spark microplate reader. ROS measurements were performed in duplicate by staining cells using the Cellular ROS Assay Kit (Deep Red) (ab186029) according to the manufacturer’s instructions (Abcam, Cambridge, United Kingdom); Ex/Em 650/675 nm. The cells were grown on glass (negative control) and PFO, F8BT, CN-PPV, SY-PPV, SO-PPV, and MEH-PPV surfaces in 5% CO2/95% O_2_ at 37°C for 48 h. ROS measurements were carried out in the dark, and subsequent to photoexcitation at 0.8 or 5 mW/mm^2^ using narrow-bandwidth Lumencor LEDs with peak emission at 390 nm for PFO, 473 nm for CN-PPV, F8BT, SY-PPV, and SO-PPV, and 548 nm for MEH-PPV.

### Electrophysiology

Whole-cell patch-clamp recordings of cultured neurons were performed at room temperature using patch pipettes (4–8 MΩ) after attaining GΩ patch seals. Traces were acquired using a HEKA EPC10 amplifier and digitizer and Patchmaster software (HEKA). The extracellular solution contained NaCl (135 mM), KCl (5.4 mM), MgCl_2_ (1 mM), CaCl_2_ (1.8 mM), HEPES (5 mM), and glucose (10 mM), and was adjusted to pH 7.4 with NaOH. The intracellular solution contained K-gluconate (126 mM), KCl (4 mM), MgSO_4_ (1 mM), CaCl_2_ (0.02 mM), BAPTA (0.1 mM), glucose (15 mM), HEPES (5 mM), ATP (3 mM), and GTP (0.1 mM) and was adjusted to pH 7.3 with KOH. The responses were amplified, low-pass–filtered at 10 kHz, digitized at 50 kHz, and analyzed with Matlab (Mathworks).

### Irradiation Procedures Patch-Clamp Recordings

Photo-irradiation of HEK239T cells and neurons grown on thin films of conjugated polymers or in the presence of CNPs were carried out on a Nikon FN1 upright microscope (Nikon Instruments) using a Spectra X LED system (Lumencor) to target the absorption spectrum maxima of the conjugated polymer under test *via* appropriate dichroic mirrors and a ×16 water immersion objective. The peak illumination wavelength was 390 nm for PFO, 473 nm for CN-PPV, F8BT, SY-PPV, and SO-PPV, and 548 nm for MEH-PPV. Gate timing was controlled by digital outputs of the HEKA patch-clamp amplifier.

### Safety

No unexpected significant hazards or risks were associated with the reported work. All animal manipulations and procedures were performed in accordance with the guidelines established by the European Community Council (Directive 2012/63/EU of 22 September 2010) and were approved by the Italian Ministry of Health.

### Statistical Analysis

Statistical tests were selected based on the data distribution. Normal distribution of the data was assessed using the D'Agostino and Pearson omnibus normality test (*p*-value = 0.05). The sample size was indicated as number of recorded cells, or culture replicates (n). The analysis was carried out with GraphPad Prism (GraphPad Software Inc.) and using custom Matlab routines (Mathworks).

## Results

### Light-Emitting Nanoparticle and Solid-State Thin-Film Conjugated Polymer Properties

**FIGURE 1:**
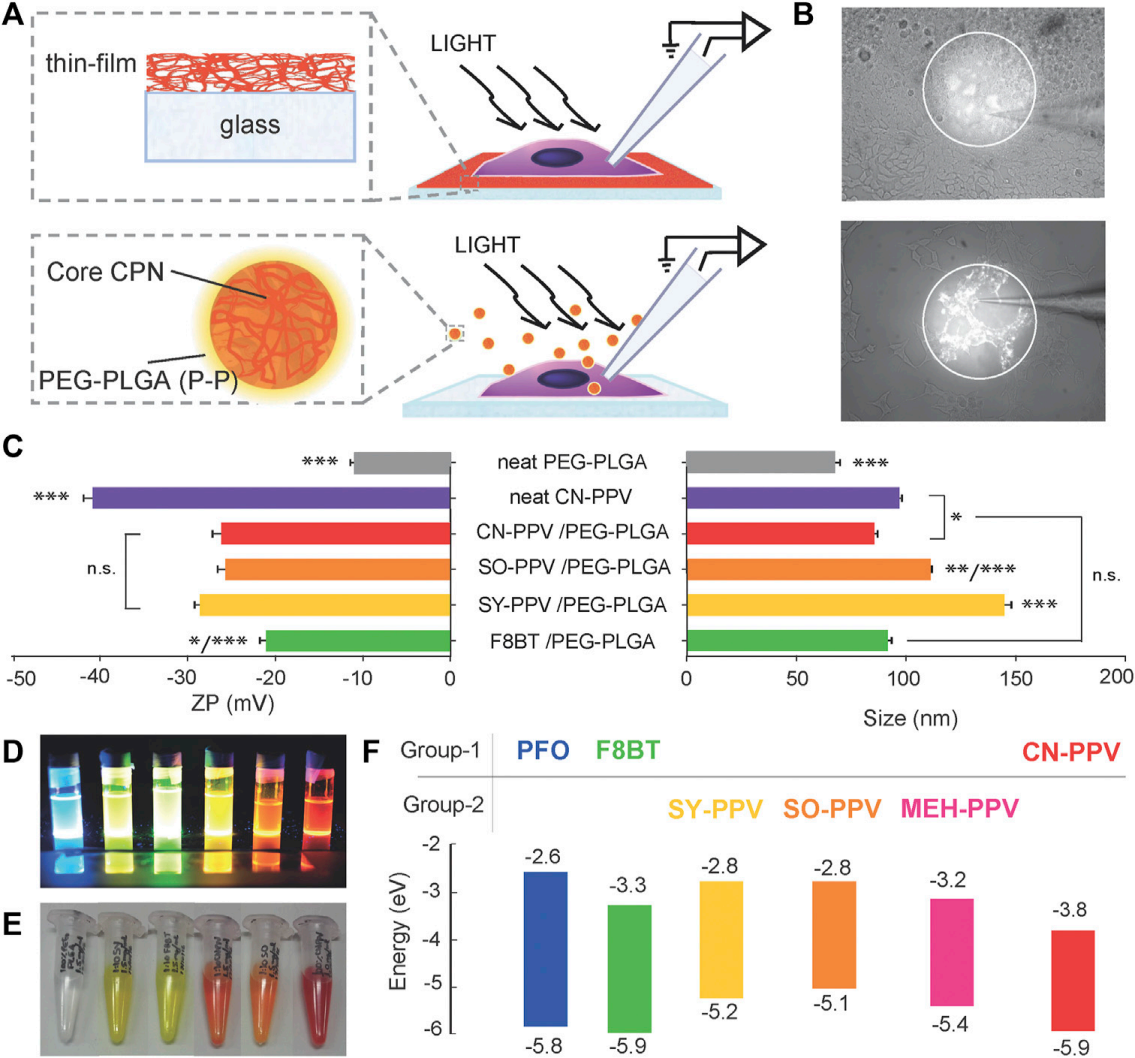
Experimental and material overview for assessment of organic conjugated polymers in solid-state thin films and nanoparticle dispersions with PEG_5k_-PLGA_55k_ amphiphilic stabilizing agent. **(A)** Patch-clamp set-up with cells grown on top of conjugated polymer thin films (top) or exposed to CNPs (bottom). **(B)** Representative images of patched cells during photo-irradiation of thin films (top) and CNPs (bottom). **(C)** Zeta potential and hydrodynamic diameters of CNPs. **(D)** Left to right: PFO, F8BT, SY-PPV, SO-PPV, MEH-PPV, and CN-PPV in THF under UV-light illumination. **(E)** Left to right: 100% PEG_5K_-PLGA_55K_, F8BT, SY-PPV, CN-PPV, SO-PPV, and 100% CN-PPV CNPs in DI-water under ambient light. **(F)** Band gap energy (Eg) for F8BT and PFO and SY-PPV, SO-PPV, MEH-PPV, and CN-PPV obtained from literature (Chua et al., 2005; Hwang and Kahn, 2005; Thompson et al., 2005; Chasteen et al., 2006; Nevil et al., 2012; Walker et al., 2014).

We selected six widely studied, commercially available light-emitting conjugated polymers that exhibit efficient photoluminescence, with optical band gap energies (*E*_g_) ranging from 2.11 to 2.8 eV. Two of the conjugated polymers were PF-derivatives (F8BT and PFO) and four were PPV-derivatives (SY-PPV, SO-PPV, MEH-PPV, and CN-PPV). For each polymer, thin films were prepared by spin coating while organic semiconductor nanoparticles (CNPs, conjugated nanoparticles) were prepared by solvent displacement. For all the preparations, cellular responses during photostimulation were recorded using patch-clamp recordings (see schematic Figures 1A,B). Key photophysical properties including ROS-generating capacity and fluorescence stability were also evaluated.

**FIGURE 2:**
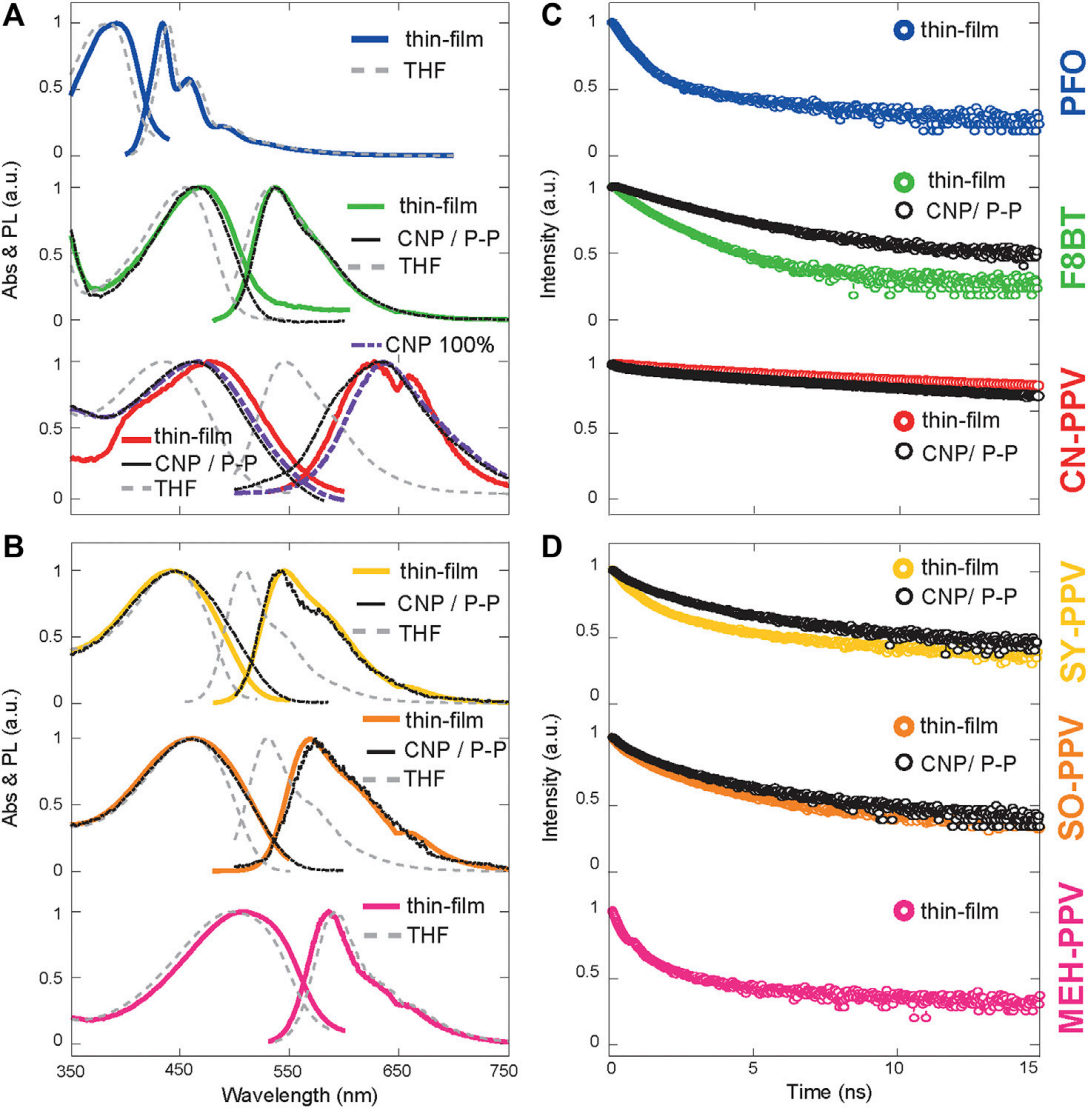
Photophysical characterization of OSC thin films and CNPs. **(A,B)** Optical absorption (Abs) and photoluminescence (PL) spectra and **(C,D)** photoluminescence decay lifetime of the six conjugated polymers in solid-state thin films and CNPs in DI-water. The spectra of the polymers in THF are also reported for comparison.

The toolkit of green to red light emitting CNPs stabilized with PEG_5K_-PLGA_55K_ was prepared via the nano-precipitation method (Abelha et al., 2017; Abelha et al., 2019). Five types of CNPs were prepared; F8BT, SY-PPV, and SO-PPV were encapsulated with PEG-PLGA, and two variants of CN-PPV nanoparticles were made with PEG-PLGA and without PEG-PLGA (100% CN-PPV, neat CNPs) (Figures 1C–F). Due to low product yield, we did not investigate PFO and MEH-PPV in nanoparticulate form. Nanoparticles containing SY-PPV and SO-PPV presented the highest product yield (≥95% by weight), followed by CN-PPV (66%), F8BT (56%), and CN-PPV without PEG_5k_-PLGA_55k_ (46%). Yields closely matched previous reports for CN-PPV and F8BT CNPs produced with PEG_5k_-PLGA_55k_ (Abelha et al., 2017; Kemal et al., 2017). Except for F8BT-CNPs, the CNPs presented a redshift in emission compared to the same conjugated polymer in tetrahydrofuran, indicating interactions between segments of the polymer chain and intramolecular energy transfer within the particles (Abelha et al., 2017). The observed red shifts were approximately 30 nm for SY-PPV, 40 nm for SO-PPV, 80 nm for CN-PPV, and ∼90 nm for 100% CN-PPV) (Figure 2A)—broadly consistent with data reported in the literature for CN-PPV and other PPV derivatives. Encapsulation of conjugated polymers within PEG_5K_-PLGA_55K_ significantly increased the CNP hydrodynamic diameter in comparison to PEG_5K_-PLGA_55K_ alone, with the increase being dependent on both the conjugated polymer chemical structure and molecular weight. SO-PPV/PEG-PLGA and SY-PPV/PEG-PLGA CNPs showed the largest hydrodynamic diameters of ∼150 nm (Figure 1C), possibly due to their higher molecular weight leading to a higher viscosity of the organic solution. Embedding CN-PPV polymer within a PEG_5K_-PLGA_55K_ matrix reduced the zeta potential of the nanoparticles compared to pure PEG-PLGA, while 100% CN-PPV CNPs were more electronegative than the PEG-PLGA counterpart, consistent with previous reports (Abelha et al., 2017).

In the form of both solid-state thin films and CNP suspensions, all polymers featured large, vibrationally resolved, absorption bands and efficient visible emission (Figure 2). Key photophysical properties of all thin films and CNPs, namely absorption (Abs) spectra, photoluminescence (PL) spectra, PL lifetime (LT), and photoluminescence quantum yield (QY) are summarized in Supplementary Tables 1–2. To gain an overview of the materials’ behavior in cell-culture systems, we studied the stability of fluorescence in a culture medium, in darkness, and under illumination. Atmospheric impurities in air and components of physiological media such as essential ions, peptides, lipids, enzymes, growth factors and other additives, and products of metabolism can play an important role in static quenching (Seidel et al., 1996; Papadopoulou et al., 2005). Although there were no observable changes in the shape of either the absorbance or photoluminescence spectrum after exposure to cell-culture media for 672 h in the dark, all materials showed a reduction in PL intensity (Supplementary Figure 1) in the range of 20%–80%. Degradation was dramatically accelerated by photo-irradiation, and the materials could be split into two groups based on their photodegradation rates. Group-1, composed of PFO, F8BT, and CN-PPV showed the largest decreases in PL intensity of 54% for F8BT, 58% for PFO, and 61% for CN-PPV when exposed to 5 h of irradiation at 0.1 mW/mm^2^ (see details in methods). Group-2, composed of SY-PPV, SO-PPV, and MEH-PPV, was moderately stable with a decrease in the photoluminescence of 11% for SY-PPV, 22% for SO-PPV, and 33% for MEH-PPV over the same observation window. The difference in degradation rates between the groups largely persisted across all tested irradiance durations and powers, as shown in the Supporting Information (Supplementary Figure 1). The two groups could also be distinguished based on their different photodegradation rates in nanoparticle suspensions (Supplementary Figure 1), with the CN-PPV- and F8BT-based nanoparticles again showing faster loss of photoluminescent intensity.

### Grouping Conjugated Polymers by ROS Generation of Thin Films and Nanoparticles

The photoexcitation of OSCs in physiological systems can lead to the formation of reactive oxygen species (ROS) that can interact with proteins, fatty acids, or nucleic acids, leading to oxidative damage. These processes can be exploited for targeted cell ablation, in which focused light delivery is employed to impair cell viability in a spatially restricted manner, for example, in cancerous tumors (Li et al., 2020). At low concentrations, ROS species act as intracellular signaling molecules, opening the possibility of using conjugated polymer nanoparticles to modulate signal cascades with subcellular resolution (Moros et al., 2018; Antognazza et al., 2019). To track photogenerated oxygen species in physiological settings, we employed a near-infrared ROS detection probe with high sensitivity to superoxide ( ${\text{O}}_{2}^{-})$ and hydroxyl (OH⋅) radicals and monitored the photosensitizing potential of the OSC films and CNPs using microglia, HEK, and HeLA cell lines as human cell models.

**FIGURE 3:**
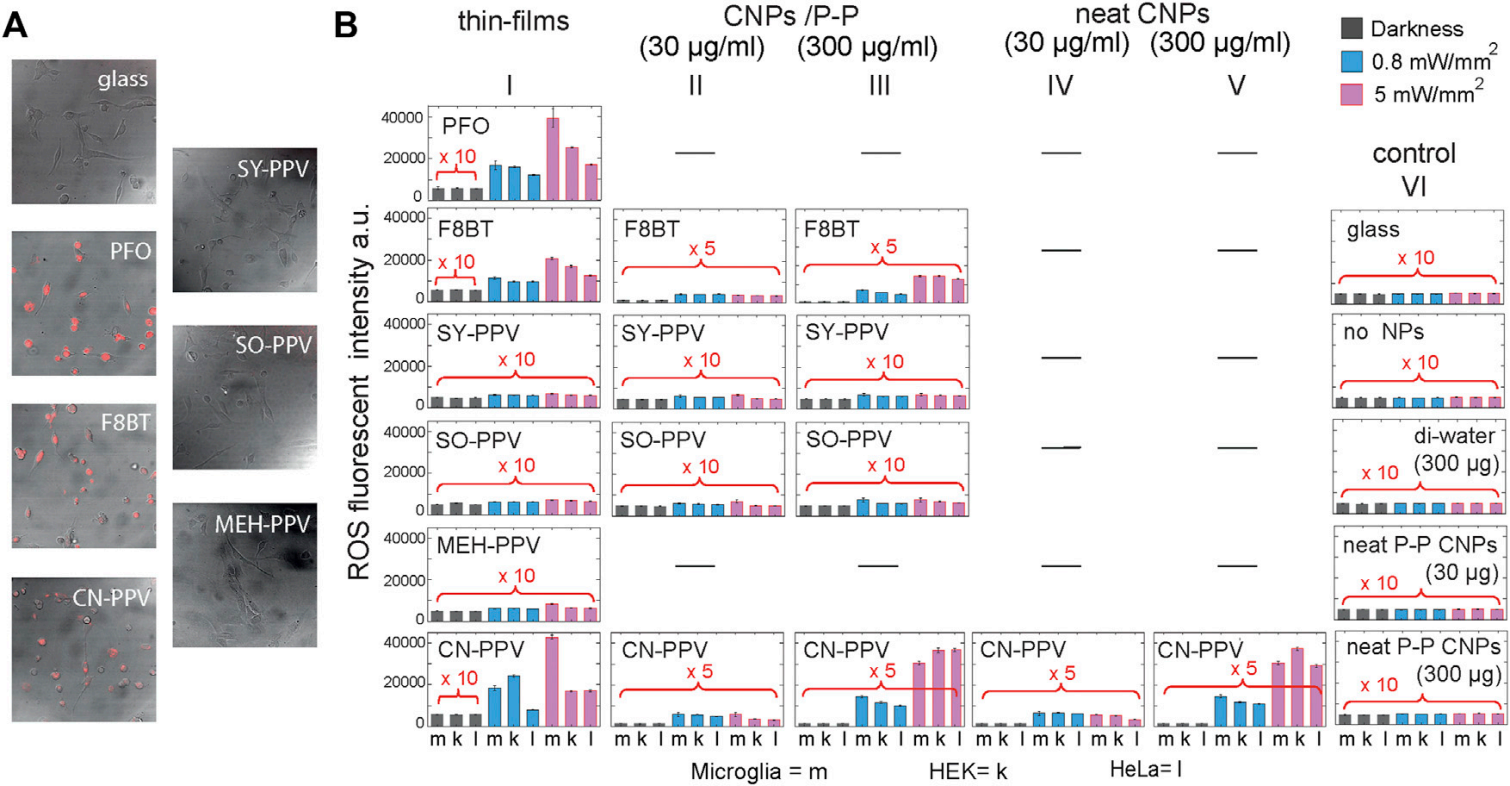
Analysis of photogenerated reactive oxygen species (ROS) by light-emitting conjugated polymers in film and nanoparticle form. **(A)** Microscope image of ROS level in red for SV40 cells grown on top of solid-state thin-film of the six conjugated polymers and glass control following irradiation. **(B)** Fluorescent assay quantifying ROS intensity in the dark (gray bars) and after irradiation with optical power of 0.8 mW/mm^2^ (blue) or 5 mW/mm^2^ (pink) at peak emission at 390 nm for PFO, 473 nm for CN-PPV, F8BT, SY-PPV, and SO-PPV, and 548 nm for MEH-PPV, for HEK239, HeLa, and SV40 cells grown on conjugated polymer thin films (column 1), CNPs encapsulated with PEG-PLGA seeded at 30 μg/ml (column 2) and CNPs seeded at 300 μg/ml (column 3), 100% neat CN-PPV nanoparticles (columns 4–5) and control glass, no CNPs, only Di-water (300 µg) and only neat PEG-PLGA (30 µg or 300 µg) (all controls, column 6). To aid in visualization, some bars have been magnified by a factor of five or 10 (red insets).

ROS generation was studied by applying a near infrared fluorescent detection assay (see methods) to live HEK239, HeLa, and SV40 cells grown on different surfaces (uncoated glass and glass coated with PFO, F8BT, CN-PPV, SY-PPV, SO-PPV, and MEH-PPV). Representative images overlaying bright-field transmission images of SV40 cells with confocal fluorescence microscopy of the fluorescent ROS indicator, following sample irradiation are shown in Figure 3A.

Figure 3B shows for the three cell lines on the various substrates of the fluorescence intensity measured after exposing the samples 1 min to 0 (dark), 0.8, and 5.0 mW/mm^2^ narrowband LED illumination (see Methods). Comparative data are also provided for cells grown in a culture medium containing 30 μg/ml and 300 μg/ml of CNPs. In the absence of illumination, cells grown on glass, cells grown on the polymer films, and cells incubated with nanoparticles showed broadly equivalent fluorescence signals, implying similar baseline ROS levels. Irradiating the cells on the polymer films with 0.8 and 5.0 mW/mm^2^ irradiation led to a significant increase in the fluorescence signal for all conjugated polymers, indicating a rise in ROS levels. However, the effect was substantially stronger for the group-1 polymers, with an irradiance of 0.8 mW/mm^2^ (blue columns) and 5 mW/mm^2^ (pink columns) of PFO, F8BT, and CN-PPV nanoparticles causing a ∼15-42-fold increase in ROS indicator intensity, compared to a range of 0.98–1.09-fold change for measurements on glass. For the group-2 polymers, increases in indicator intensity were less than three-fold. The cells incubated with CNPs exhibited broadly similar behavior, and samples incubated with 300 μg/ml F8BT and CN-PPV-based nanoparticles showed respective increases in assay fluorescence of 6.2-19 (F8BT/PEG-PLGA), 6.6–23.9 (CN-PPV/PEG-PLGA), and 7.2–24 (CN-PPV neat), compared to 1.06–1.14 for cells seeded with pure PEG-PLGA nanoparticles, while those incubated with 300 ug SY-PPV/PEG-PLGA and SO-PPV/PEG-PLGA showed weaker increases in the range of 1.26–1.44 and 1.24–1.61, respectively. (No data are provided for cells incubated with PFO-based nanoparticles since we could not obtain unaggregated particles using the preparation method described). Weaker increases in the fluorescence signal were observed at a 30 μg/ml incubation concentration.

In all cases, the response to photostimulation was found to be broadly independent of the cell types used in the present study. Microglial (SV40), epithelial (HEK293), and human epithelial adenocarcinoma (HeLa) cells all presented ROS increases upon photoexcitation when in contact with OSC thin films and nanoparticles. We concluded that all the tested materials lead to significant ROS formation (in both thin-film and nanoparticulate form) when illuminated at their peak absorption wavelengths, with polymers containing fluorene and cyano-terephthalylidene generating the highest ROS levels.

### Divergent Responses of Cell Membrane Potential to Photoexcitation

**FIGURE 4:**
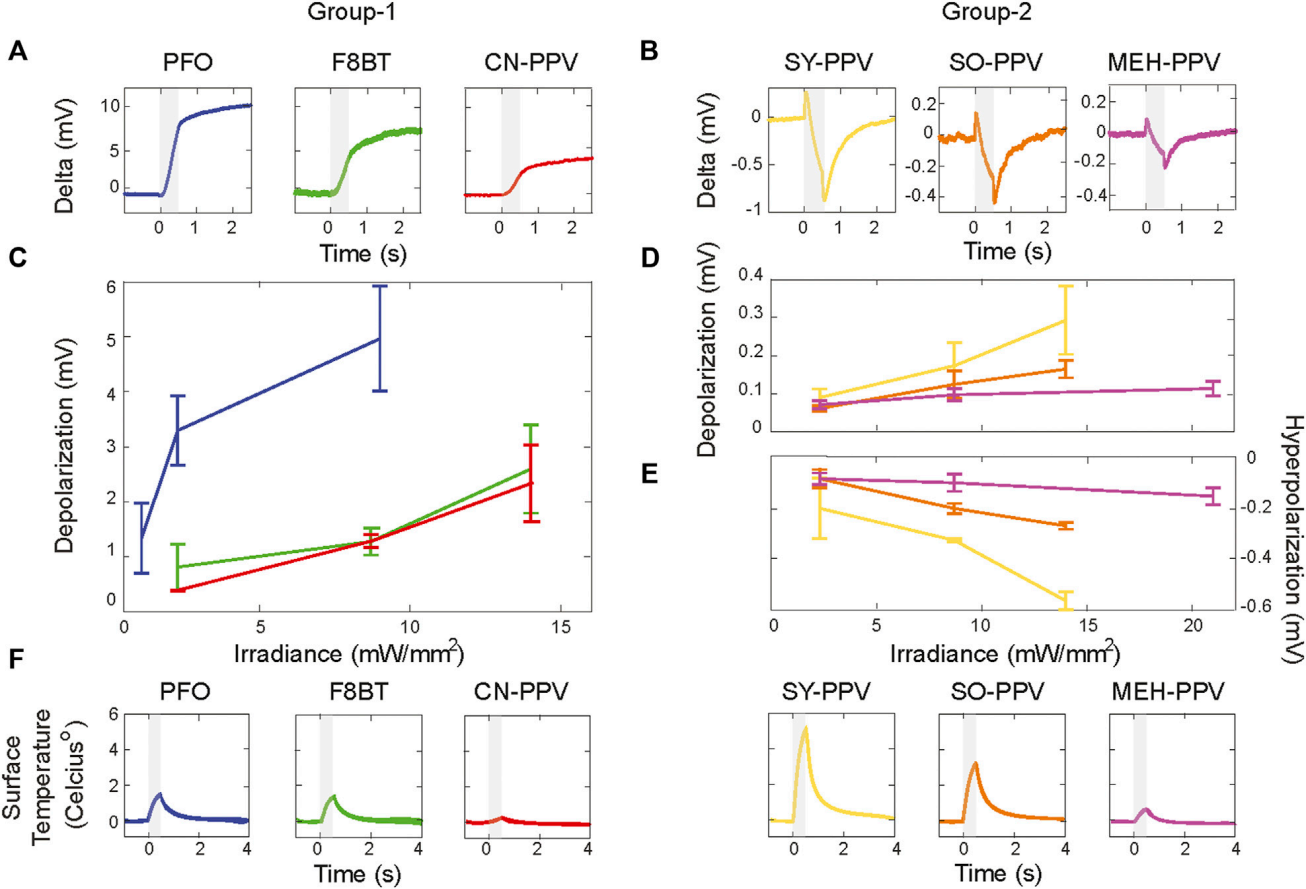
Plurality of response types of the cell membrane potential of HEK293T cells following photon absorption in adjacent semiconducting polymers. Cell responses recorded for each material at different light intensities using narrowband filtered LEDs to match excitation wavelength to the absorbance peak of thin film (390 nm for PFO, 473 nm for CN-PPV, F8BT, SY-PPV, and SO-PPV, and 548 nm for MEH-PPV). Two broad types of responses are observed in response to 500 ms illumination (gray areas highlight the light irradiation period): group-1 materials PFO, F8BT, and CN-PPV **(A)** show irreversible depolarization which extends beyond the light period; group-2 materials (SY-PPV, SO-PPV, and MEH-PPV) **(B)** show reversible multiphasic stimulations. Representative traces are depicted for the highest employed irradiation power, t = 0 corresponds to light onset. **(C–E)**. Amplitude during illumination versus irradiation power and material (mean ± SEM, N = 2–6 cells per tested irradiance). **(F)** Surface temperature measured during 500 ms illumination reveals the largest temperature shifts for SY-PPV films, followed in decreasing order by SO-PPV, PFO, F8BT, MEH-PPV, and CN-PPV. The irradiance used for each film corresponds to the highest power tested during cellular photostimulation.

Next, we investigated the effects of photo-stimulation on the cell membrane potential of HEK293T cells. This cell line does not express the ion channels required to generate rapid electrical currents across its plasma membrane, allowing observations of effects that translate across mammalian cell types. We first cultured HEK293T cells on coverslips that had been spin-coated with one of the six polymers. Forty-eight hours after plating the cells, light-evoked responses were assessed by the whole-cell patch-clamp, in current-clamp (I = 0) configuration (Figure 4). The conjugated polymers were excited at their respective absorbance peaks (see Methods) for 500 ms, allowing for direct comparison to our previous work on photothermal stimulations with polythiophene-type materials (Martino et al., 2015; Feyen et al., 2016) (Figures 4A,B). Two types of responses could be identified across the materials, with a grouping of responses following the ROS production capacity of the materials inferred from Figure 1B. Photostimulation of PFO, F8BT, and CN-PPV led to a rapid depolarization of the membrane potential which did not reverse when illumination ceased (group 1; Figure 4A). The photo-response of cells grown on the group 2 materials (SY-PPV, SO-PPV, and MEH-PPV) was reversible and characterized by an initial depolarization followed by sustained hyperpolarization during illumination (group 2; Figure 4B). For both response types, the magnitude of the membrane potential variation during light stimulation scaled positively with the irradiance level (Figures 4C–E).

For group-2 OSCs, the kinetics and waveform of the membrane potential changes (initial depolarization followed by a sustained membrane hyperpolarization) qualitatively matched those we have previously reported for HEK293T cells grown onto polythiophene-type thin films (Martino et al., 2015; Feyen et al., 2016). We have previously shown that this multiphasic membrane response to intense and prolonged light stimuli is attributable to thermal stimulation following non-radiative recombination of photo-excited electrons and holes (Martino et al., 2015). On the cellular side, the response is mediated by increased membrane capacitance, the temperature dependence of the transmembrane potential, and temperature-mediated activations of membrane currents. To directly assess the relationship between the evoked responses and the peak surface temperature elicited by photostimulation, we measured the temperature variation at the film surface for each of the tested materials using the calibrated pipette resistance method (Yao et al., 2009). Surface temperature measurements were carried out using the highest irradiance power tested per film during cellular photostimulation*.* Light-evoked increases in surface temperature were present for all materials (Figure 4F) with CN-PPV films showing the lowest (0.28 ± 0.02°C) and SY films the largest (5.29 ± 0.01°C) temperature rise. The variance in surface temperature measured across the materials can be largely accounted for by the absorbance of the OSC films and the employed irradiance power (Supplementary Figure 2). In agreement with our previous reports, we found that the magnitude of depolarization and hyperpolarization of cells plated on the group-2 materials scales as a function of the measured surface temperature increase (see Supplementary Figure 3; SO-PPV (3.34 ± 0.01°C), SY-PPV (5.29 ± 0.01°C), and MEH-PPV (0.71 ± 0.02°C)). In contrast, given the intermediate to low temperature increase measured for PFO (1.54 ± 0.01°C), F8BT (1.45 ± 0.02°C), and CN-PPV films (0.28 ± 0.01°C), we deemed it unlikely that thermal membrane ablation contributes to the depolarizations observed with group-1 OSCs.

**FIGURE 5:**
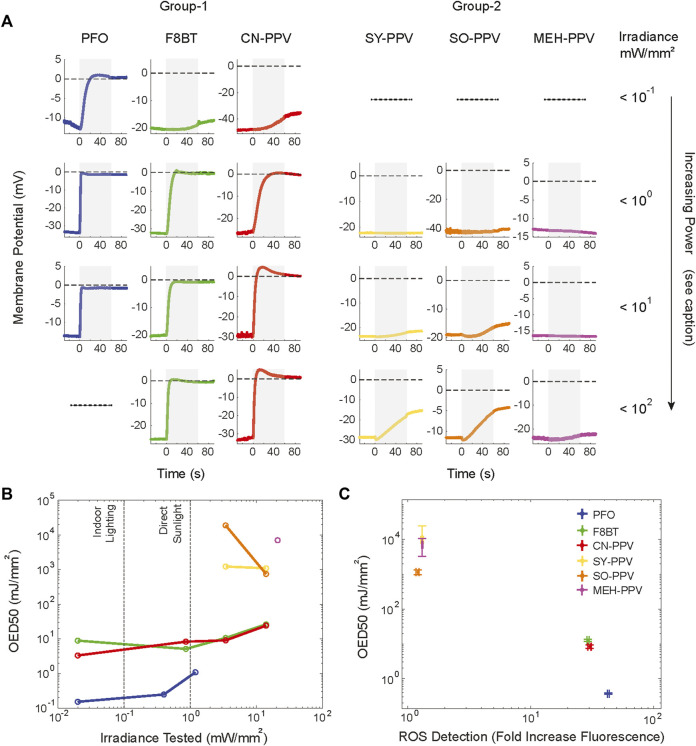
Kinetics and waveform of the effects of photostimulation of cells grown on group 1 materials and progressive changes of the photo-response of cells grown on group 2 materials. Membrane potentials of HEK293 cells were recorded during 1-min photoexcitation at multiple irradiance levels. **(A)** Mean traces of the membrane potential response during 1-min illumination at increasing irradiances (N = 4–10 cells per material), light onset at t = 0. Tested irradiances per material (mW/mm^2^); PFO (0.02,0.4,1.2; 390 nm), F8BT (0.02,0.86,3.44,14; 473 nm), CN-PPV (0.02,0.86,3.44,14; 473 nm), SY-PPV (0.86,3.44,14; 473 nm), SO-PPV (0.86,3.44,14; 473 nm), and MEH-PPV (0.86, 5.7, and 21; 548 nm). **(B)** Scatter plot of mean OED_50_ as a function of irradiance and material used. **(C)** Observation of an inverse power law relationship between measured ROS evolution (fold increase in reporter fluorescent intensity) and mean OED50 value obtained across irradiance powers per material (OED50 [mJ/mm^2^] mean ± SEM; PFO (0.37 ± 0.02), F8BT (12.69 ± 0.81), CN-PPV (8.35 ± 0.93), SY-PPV (9.6x10^3^ ± 1.52x10^3^), SO-PPV (1.15x10^3^ ± 0.18x10^3^), and MEH-PPV (6.98x10^3^ ± 3.76x10^3^).

An interesting question is how far a generated ROS can diffuse before decaying via a unimolecular process or being scavenged by a target molecule. Given that all tested materials generated ROS, albeit to substantially varying levels, we sought to assess if stimulus duration or power would modulate the membrane response. We hypothesized that under prolonged illumination, ROS species could accumulate to high concentrations in the cleft between the cell membrane and the film, increasing the probability of interaction events with membrane components, and thus potentially leading to an alteration of the photo-mediated response. To analyze the consequences of prolonged illumination and better measure the kinetics of the depolarizations observed with the group-1 materials, PFO, F8BT, and CN-PPV films, we employed 1-min light stimuli at varying irradiances, while continuously recording cellular membrane potential (Figure 5A). For the group-1 materials, we observed that prolonged illumination leads to a rapid depolarization of the membrane potential with a response plateau near 0 mV, indicating a loss of membrane integrity and a resulting drop in membrane resistance. For PFO, this response was apparent during 1-min stimulation with an irradiance power density of 0.02 mW/mm^2^, which is close to typical daytime irradiances. The 0-mV plateau response became observable within the 1-min illumination time for F8BT and CN-PPV for irradiances ≥0.8 mW/mm^2^.

Interestingly, during prolonged illumination at high irradiance levels (>1 mW/mm^2^), the membrane potential responses of cells grown on the group 2 OSCs displayed irreversible depolarization components in their responses (Figure 5A; bottom rows). Although shorter 500-ms illumination resulted in transient and reversible changes in membrane potential (Figure 4A), prolonged illumination at higher irradiance levels led to irreversible depolarization, similarly to the behavior observed for cells grown on PFO, F8BT, and CN-PPV. To directly compare the kinetics of the irreversible depolarization across irradiance power and materials, we quantified the cumulative optical energy delivered at the time the membrane potential reached 50% depolarization relative to the resting potential and 0 mV (OED_50_). The values were extracted based on empirical observation or linear extrapolation of the observed response during illumination. Recordings with depolarizations of less than 5% of RMP were considered to show a safe response and were not included in OED_50_ analysis. The OED_50_ value metric is intended to capture the phototoxic potential for different material classes, and to provide an indication of a safe operating range. The OED_50_ value calculated per material and irradiance is reported in Figure 5B. The cells grown on PFO showed the fastest rates of depolarization, and correspondingly the lowest OED_50_ values. The OED_50_ values of the group-1 materials (PFO, F8BT, and CN-PPV) were separated by several orders of magnitude from the group-2 materials (SY-PPV, SO-PPV, and MEH-PPV). Furthermore, group-2 materials only displayed observable OED_50_ at irradiances >0.86 mW/mm^2^ for SO-PPV and SY-PPV, and MEH-PPV for irradiance powers >5.7 mW/mm^2^, yielding upper bound estimates for the safe operating window of these materials. Response amplitudes in mV across the tested conditions are reported in Supplementary Table 3. A ‘close-up’ of the initial 10 s of a cell grown on SY-PPV and irradiated with 14 mW/mm^2^ light is presented in Supplementary Figure 4. We found that the mean OED_50_ measured across irradiance powers shows an inverse power law relationship to the ROS generation capacity of the films inferred from the reporter fluorescence in HEK293 cells (Figure 5C; *linear regression fit* log(*OED*_*50*_) vs*.* log*(fold increase in ROS fluorescence); r* ^*2*^ *= 0.9143, p-value = 0.003*), denoting the observed positive relationship between ROS generation capacity of a material and the rate at which it induces membrane depolarization.

We next investigated the spectral dependence of the irreversible stimulations observed with PFO and F8BT films. For both materials, cell membrane responses increased as the excitation wavelength was shifted toward the respective absorption maxima (Supplementary Figure 5). Focusing on F8BT, we carried out two series of experiments to directly assess the involvement of oxygen in mediating the responses. In the first one, we coated a 2 µm layer of SU8 photoresist onto F8BT, reducing oxygen availability and generating a physical barrier for quenching generated radical species. This fully abolished the photo-response. Given previous reports on singlet oxygen production by F8BT in water (Spada et al., 2018), we assessed whether the introduction of the singlet oxygen scavenger sodium azide (NaN_3_) in the extracellular solution would slow the kinetics of the irreversible depolarization. Indeed, the addition of 100 mM NaN_3_ significantly slowed cellular depolarizations with respect to a NaN_3_-free control, as reflected by the higher OED_50_ values of 16.67 ± 5.5 mJ/mm^2^ versus 4.08 ± 2.3 mJ/mm^2^ for recordings with and without NaN_3_, respectively (see Supplementary Figure 5; Mann–Whitney *U*-test, *p* < 0.01). The results indicate that singlet oxygen production, following photoexcitation of F8BT films, contributes to the measured cellular membrane response. Together with ROS detection, which has the sensitivity to superoxide and hydroxyl evolution, multiple photochemical processes may be active at the polymer–cell interface. On the basis of the OED_50_ values, we consider group-2 materials preferable for imaging applications due to reduced ROS generation. In contrast, notwithstanding their high fluorescence quantum yields (see Supplementary Table 1), the low OED_50_ values of group 1 suggest suitable applications in cell ablation, sterilization, or photocatalysis.

### Dose-dependent Light-Induced Depolarization in HEK293 and Irreversible Depolarization and Firing in Primary Neurons by CNPs

**FIGURE 6:**
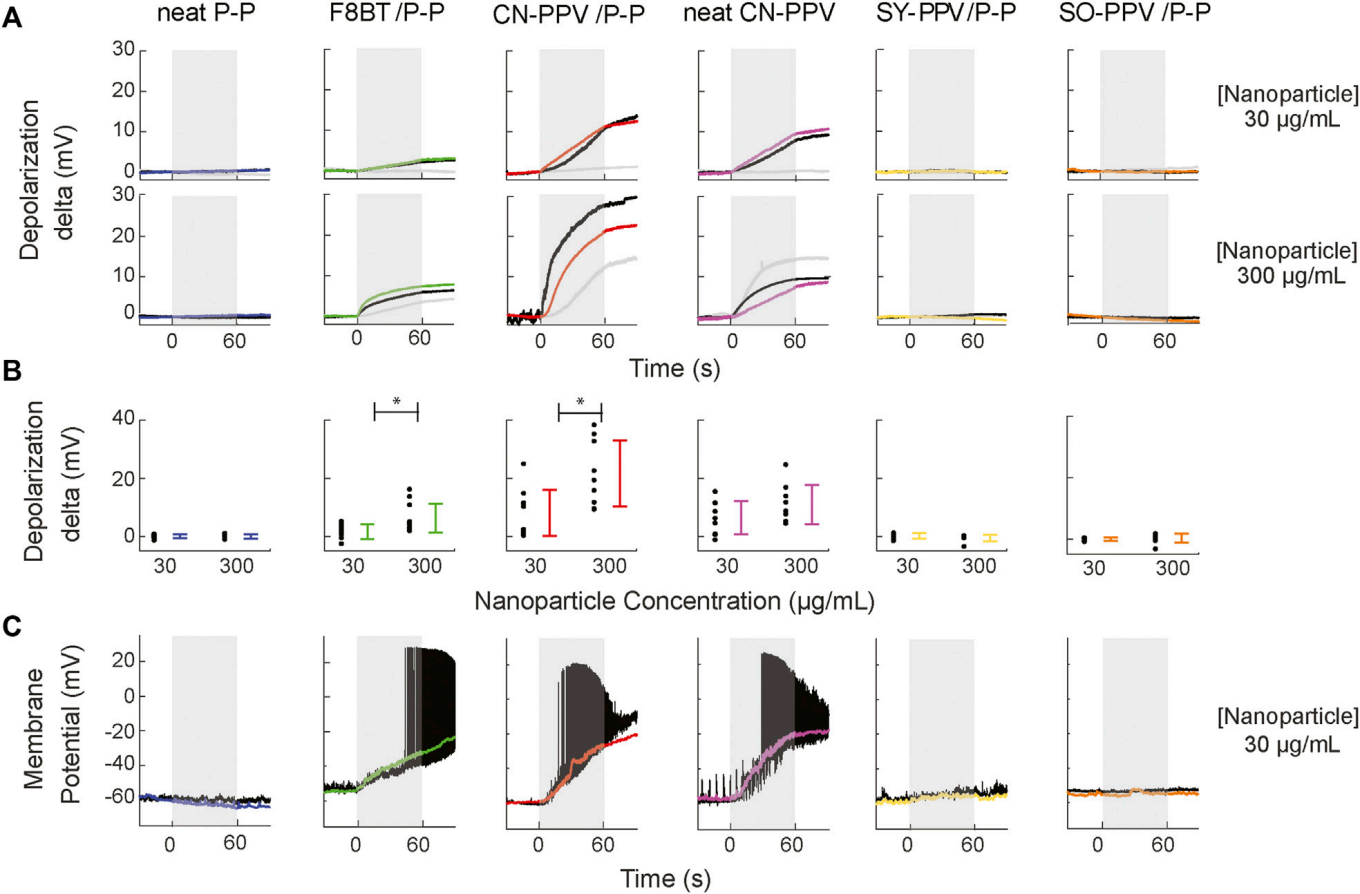
Membrane potential response to photoexcitation of HEK293 and primary neurons incubated with conjugated polymer nanoparticles. **(A)** Traces of the mean responses of HEK293 cells per experimental condition, with gray, black, and colored traces for 0.8, 3.4, and 5 mW/mm^2^ (473 nm), respectively N = 3–4 cells per irradiance. Recordings in top and bottom rows correspond to cells incubated with CNP concentrations of 30 and 300 μg/ml, respectively. **(B)** Concentration-dependence of depolarization of HEK293 cells. **(C)** Depolarization elicited by photostimulation of primary neurons incubated with CNPs. Black traces show representative traces of neuronal responses; colored lines show the mean response low-pass filtered at 100 Hz (N = 3–4 neurons per condition).

We next checked the functional consequences of CNP illumination on the membrane electrical properties. Using patch-clamp recordings, we sought to assess whether the OED_50_ values obtained in thin films hold predictive validity for the phototoxicity of the OSCs in nanoparticle form. We plated HEK293T cells on glass coverslips and, 12 h after cell seeding, incubated the cells for 24 h with either 30 or 300 μg/ml of CNPs. Using whole-cell patch-clamp recordings, we tracked the membrane potential response to illumination at various power densities of incident light (0.8, 3.4, and 5 mW/mm^2^; 1 min). In response to light, two distinct responses were readily observed. While cells incubated with PEG-PLGA controls, SO-PPV/PEG-PLGA or SY-PPV/PEG-PLGA nanoparticles were broadly unaffected by light, the membrane potentials of cells incubated with F8BT/PEG-PLGA, CN-PPV/PEG-PLGA, or 100% CN-PPV nanoparticles displayed depolarizations that were time-locked to the light onset (Figure 6A).

No consistent relationships could be identified between light intensity and cell responses in this data set (Figure 6A), likely reflecting a variable quantity of membrane bound and internalized CNPs by different cells. To quantify the depolarization effect across the tested CNP concentrations, we grouped the recorded cells across irradiance per CNP type. This analysis captured a dose-dependent increase in depolarization for F8BT/PEG-PLGA and CN-PPV/PEG-PLGA nanoparticles (Mann–Whitney *U*-test; *p* < 0.05, depolarizations 30 vs. 300 μg/ml) and a similar trend for CN-PPV nanoparticles (mean ± std; 6.02 ± 5.28 mV vs. 10.39 ± 7.91, Mann–Whitney *U*-test; *p* = 0.19) (Figure 6B). Importantly, for all three CNPs that induced a depolarizing response, the membrane potential did not recover in the 30-s after illumination ended. The materials with the lowest OED_50_ values in thin-film form also modulated the cell membrane potential in a similar irreversible fashion when they were in nanoparticle form.

Given their nanoscale size and propensity for uptake by cells, a potential application of CNPs is in the labeling and manipulation of neural circuits. We examined how the depolarization observed in cell lines would translate to electrically excitable neuronal cells. To this end, we cultured primary hippocampal neurons on glass coverslips and, after maturation of the neural network, incubated these cells with CNPs and recorded them in whole cell configuration during 1-min light stimulation. As with HEK293 cells, this illumination protocol had no effect on the membrane potential of neurons grown in culture media containing PEG-PLGA control CNPs, or group-2 CNPs (SY-PPV/PEG-PLGA or SO-PPV/PEG-PLGA). By contrast, upon illumination of neurons bearing F8BT/PEG-PLGA, CN-PPV/PEG-PLGA, or non-encapsulated CN-PPV nanoparticles, the neuronal membrane potentials rapidly depolarized, resulting in action potential discharges, a progressive decrease in action potential amplitude, and reductions in the membrane resistance (Figure 6C). We, therefore, suggested that SY-PPV/PEG-PLGA and SO-PPV/PEG-PLGA could be further investigated for imaging applications, including retrograde labeling of neural circuits. F8BT/PEG-PLGA and CN-PPV/PEG-PLGA could be explored in a similar context and be further exploited *in vivo* for their ability to lesion neural circuit elements through focused light illumination.

## Conclusion

Using a multi-level comparison involving both thin films and nanoparticles, ranging from photophysical to membrane potential measurements, we have investigated the processes caused by the photostimulation of OSC materials in the vicinity of cellular plasma membranes. Two types of membrane responses were observed: (i) reversible multiphasic photothermal responses and (ii) irreversible depolarization driven by photosensitization of molecular oxygen. Using thin-film OSCs we further found that—under intense and/or prolonged illumination—rapid photothermal effects can give way to irreversible processes, which are likely driven by accumulation of photogenerated ROS that disrupt membrane integrity, yielding progressive, and irreversible depolarizations. In the case of F8BT and CN-PPV, these irreversible membrane modifying properties are also observed when they are formulated as PEG_5k_-PLGA_55k_ encapsulated CNPs. In contrast, formulation of SO-PPV and SY-PPV as nanoparticles resulted in “safer” ROS levels under illumination that did not irreversibly change cellular polarization.

In no case, the photosensitized stimulation was found to reverse in the recorded time window. From recordings on HEK293, we found that the depolarization amplitude is dependent on both light intensity and nanoparticle concentrations. In neurons, stimulation led to intense action potential firing. The amplitude of the action potentials decreased over the illumination period, before firing of the cells was silenced. The persistence of the electrophysiological responses in HEK293 and neurons (progressive depolarization), suggests that key mechanisms are generalizable across cell types. The observed phenomena are likely to be mediated by the modulation of transmembrane currents and the fundamental electrical properties of the cell membrane (e.g., lipid peroxidation, which is known to generate ion and water-conducting pores in the plasma membrane). Previous reports on photosensitizers including photofrin II, rose Bengal, and protoporpyrin IX have yielded similar results; illumination gave rise to depolarization, inactivation of calcium-dependent K^+^ channels, and increased leak conductances (Killig et al., 2001). Action potential firing and progressive decrease in action potential amplitude due to the progressive membrane depolarization have been described in the case of PPa-sensitized production of singlet oxygen in neurons (Breitenbach et al., 2010). It is interesting to note that group-1 materials possess the lowest HOMO values: −5.8 eV for PFO, −5.9 eV for F8BT, and −5.9 eV for CN-PPV compared to −5.2 eV for SY-PPV, −5.1eV for SO-PPV, and −5.4 eV for MEH-PPV. The group-2 materials, SY-PPV, SO-PPV, and MEH-PPV, show higher stability and lower ROS levels. Hence, molecules of materials in group-1 (PFO, F8BT, and CN-PPV) may be easily ionized (oxidized) at their surface from bio-environmental contacts compared to the other polymers studied.

From an application perspective, we have generated a multicolor toolset of PEG-PLGA encapsulated nanoparticles presenting high processing yields, with diameters between 85 and 145 nm, and emissions spanning the visible spectrum. Their respective properties are ideally suited for a range of biomedical applications including, but not limited to, subcellular scale imaging applications, drug delivery, cellular ablation, and localized ROS production. The behavior of PFO, F8BT, and CN-PPV films as effective ROS generators, potentially opens up applications of these OSCs as sterilization agents, in biodegradation, treatment of large area infections, or flow-cell photocatalysis applications using low-dose irradiation. In particular, PFO stands out, having obtained an OED_50_ value of 0.37 mJ/mm^2^ and yielding depolarizations at irradiance values equivalent to common daytime and indoor ambient light conditions. In separate work, we have used the same light-emitting polymers in active devices for photo-stimulation of opsin-expressing neurons (Matarèse et al., 2019) and for optical brain–imaging (Matarèse et al., 2020) with evidence of their use in biocompatible organic light-emitting diodes (Matarese, 2021). This work provides evidence that group-2 light-emitting polymers, such as SY-PPV and SO-PPV, are more suitable for the next generation of passive photoemitters, due to their reduced ROS-mediated side effects.

While for some applications, such as neural prosthetic applications or live-cell imaging, nanoparticles should remain intact over long periods of time, in other applications, such as photodynamic therapy, the general concept is that the nanoparticles should be biodegradable. Thus, an investigation of nanoparticle stability under light irradiation in aqueous systems needs to be investigated more closely for each individual polymer to identify the suitability toward clinical *in vivo* applications, and the extent to which the photo-mediated degradation can be exploited to beneficial ends.

In sum, broad evaluation of photonics tools is critical to obtaining a stable and specific biological interface. The non-linear response properties of cellular membranes to varied illumination duration and power which we have observed in this study underscore the potential for “off-target” effects of photoexcitation, which should be accounted for in experimental design and medical applications. At the same time, the tested polymers and CNP counterparts showed reliable stimulation of cellular membrane potentials, the ability to generate ROS, and robust emissive properties over prolonged aqueous exposure, suggesting many avenues for tailored applications of the materials characterized herein.

## Data Availability

The original contributions presented in the study are included in the article/Supplementary Material; further inquiries can be directed to the corresponding authors.
