# Supplementary material for: Photosensitized and Photothermal Stimulation of Cellular Membranes by Organic Thin Films and Nanoparticles
Source: Front Bioeng Biotechnol. 2022 Jul 7;10:932877. doi: 10.3389/fbioe.2022.932877 (PMC9302485; doi:10.3389/fbioe.2022.932877)
Supplement: Supplementary file 1 [file DataSheet1.PDF]

SUPPLEMENTARY INFORMATION

Supplementary Table 1. The main photophysics properties, absorption (Abs), photoluminescence (PL), lifetime (LT) and quantum yield (QY) of the conjugated polymers in their solid-state thin-film form.

	Material	*Abs max (nm)*	*PL max (nm)*	*Optical Bandgap (eV)*	*PLQY (%)*	*PL Lifetime (ns)*
Group-I	PFO	392	434	2.8	55	0.46
	F8BT	469	536	2.35	51	0.64
	CN-PPV	478	628	2.14	35	5.97
Group-II	SY-PPV	441	545	2.3	17	0.679
	SO-PPV	460	569	2.2	19	0.58
	MEH-PPV	506	585	2.11	29	0.41

Supplementary Table 2. The main photophysic properties, absorption (Abs), photoluminescence (PL), lifetime (LT) and quantum yield (QY) of the conjugated polymers in suspended nanoparticle form.

	Material	*Abs max (nm)*	*PL max (nm)*	*PLQY (%)*	*PL lifetime (ns)*
Group-I	F8BT / PEG-PLGA	464	535	56	1.14
	CN-PPV / PEG-PLGA	462	632	39	3.19
	CN-PPV 100%	467	636	38	3.35
Group-II	SY-PPV / PEG-PLGA	446	540	21	0.84
	SO-PPV / PEG-PLGA	460	575	20	0.82

Supplemntary Table 3. Employed irradiances for assessment of OED50, and measure membrane response expressed as maximum amplitude of depolarization (mV) during light irradiation relative to baseline (mean ± std). NT, not tested.

	**lambda (nm)**	**Irradiance 1-4 (mW/mm2)**	**Irradiance 1 $<10^{-1}$**	**Irradiance 2 $<10^{0}$**	**Irradiance 3 $<10^{1}$**	**Irradiance 4 $<10^{2}$**
**PFO**	390	0.02; 0.4; 1.2; N/T	13.76 mV; n=1	33.81 mV; n=1	13.85±7.25 mV; n=2	*N/T*
**F8BT**	473	0.02; 0.86; 3.44; 14	2.35±3.3 mV; n=2	34.62±14.04 mV; n=4	20.63±6.3 mV; n=3	27.5 mV; n=1
**CN-PPV**	473	0.02; 0.86; 3.44; 14	10.21 mV; n=1	27.09±5.53 mV; n=2	34.45±5.67 mV; n=3	38.19 mV; n=1
**SY-PPV**	473	N/T; 0.86; 3.44; 14	*N/T*	0.82±0.31 mV; n=3	2.27±1.30 mV; n=3	11.18 mV; n=1
**SO-PPV**	473	N/T; 0.86; 3.44; 14	*N/T*	1.05 mV; n=1	3.36±3.12 mV; n=5	6.86±0.85 mV; n=4
**MEH-PPV**	548	N/T; 0.86; 5.68; 21	*N/T*	0.62±0.19 mV; n=2	0.84±0.28 mV; n=4	2.11±1.62 mV; n=4

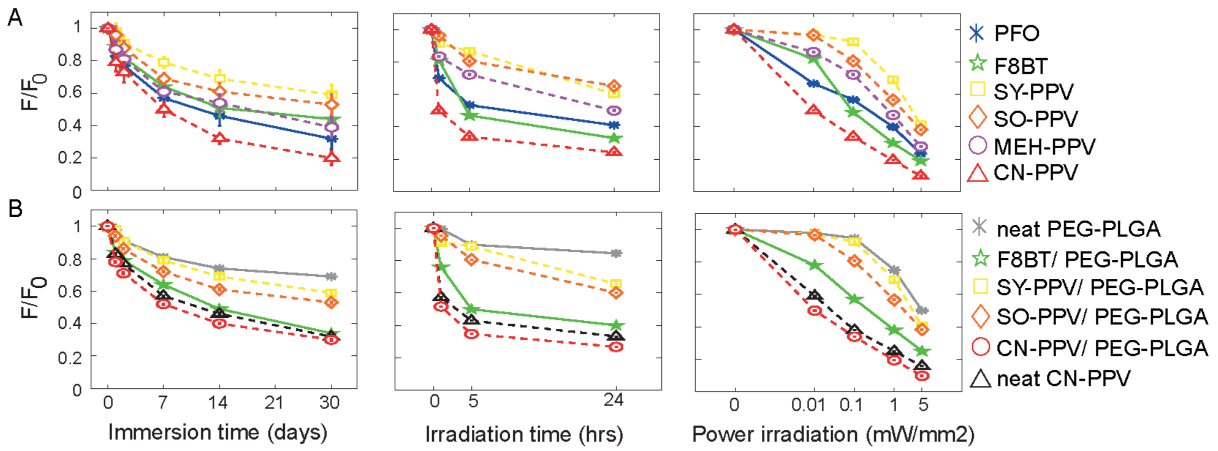

Supplementary Figure 1. Stability of fluorescence intensity (luminance F/F0) of cast thin films (a) and nanoparticles (b). *Left Column:* Fluorescence intensity variations as a function of incubation time in culture medium in darkness. *Middle Column:* Fluorescence intensity variations as a function of irradiance time (at 0.2 mW/mm^2). *Right Column:* Fluorescence intensity variations as a function of irradiance power, for equal irradiance duration (3 hrs).

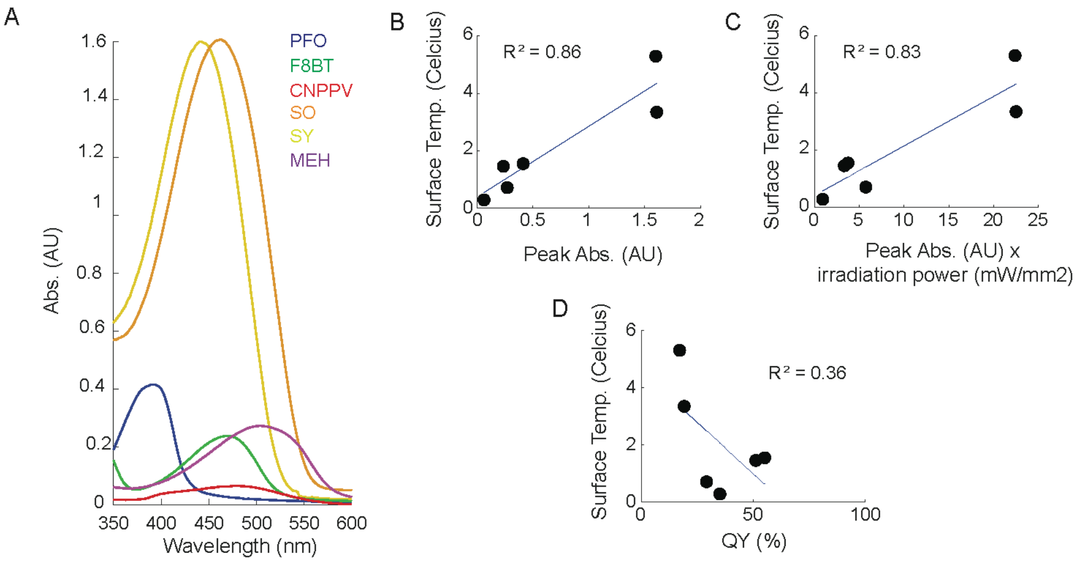

Supplementary Figure 2. Measured surface temperature as a function of absorbance. (a) Absorbance spectrum of cast-thin films. (b) Scatter plot of peak absorbance vs surface temperature per material, yielding a linear fit with r-squared = 0.86. (c) Scatter plot of surface temperature versus estimate of absorbed energy (c) Scatter plot of QY of photoluminescence versus surface temperature per material.

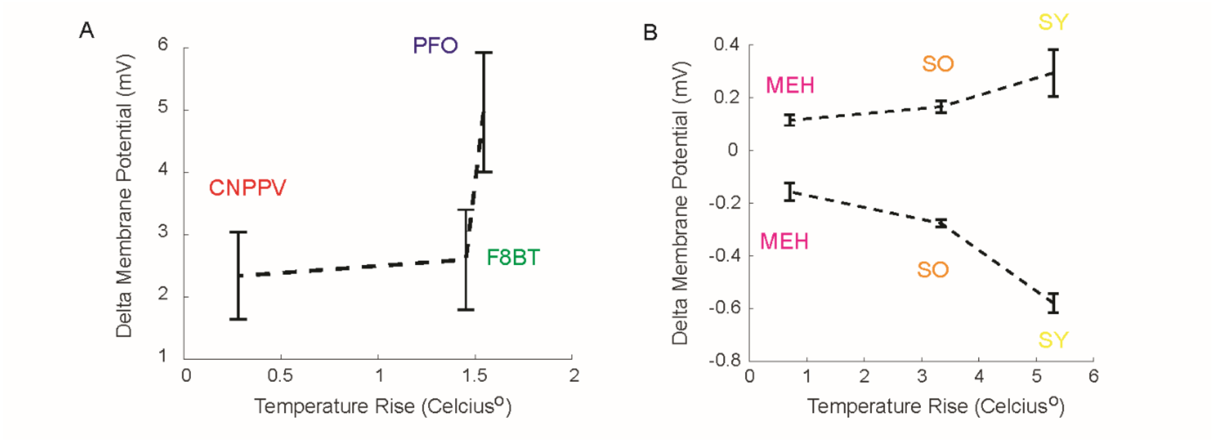

Supplementary Figure 3. Response amplitudes (mV) *versus* surface temperatures (degrees Celsius) measured during 500ms equal irradiation power density (mW/mm^2); 9 (PFO), 14 (F8BT, CN-PPV,SO-PPV,SY-PPV), and 21 (MEH-PPV). Showing depolarization amplitudes (a) and depolarization alongside hyperpolarization amplitudes (b).

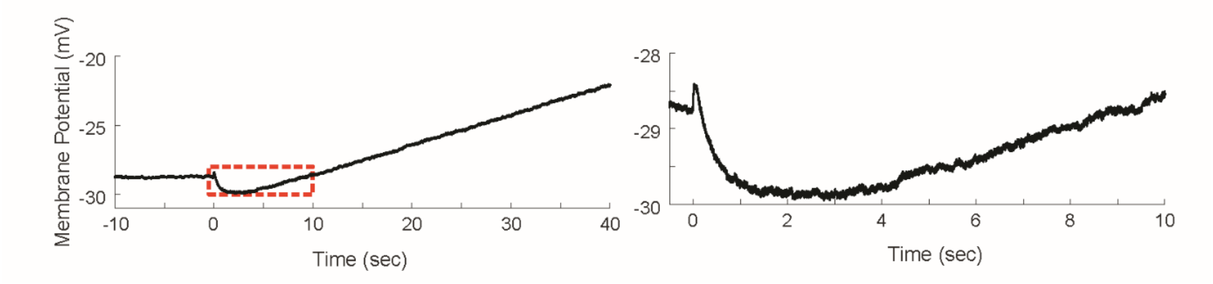

Supplementary Figure 4. Representative patch-clamp recording trace of a HEK293 cell plated on SY-PPV and subjected to 1 min irradiance at 14 mW/mm^2 (light onset at t=0). The transition from a reversible photothermal response to sustained depolarization is apparent. The red box (left) indicates the part of the plot given in an expanded timescale (right).

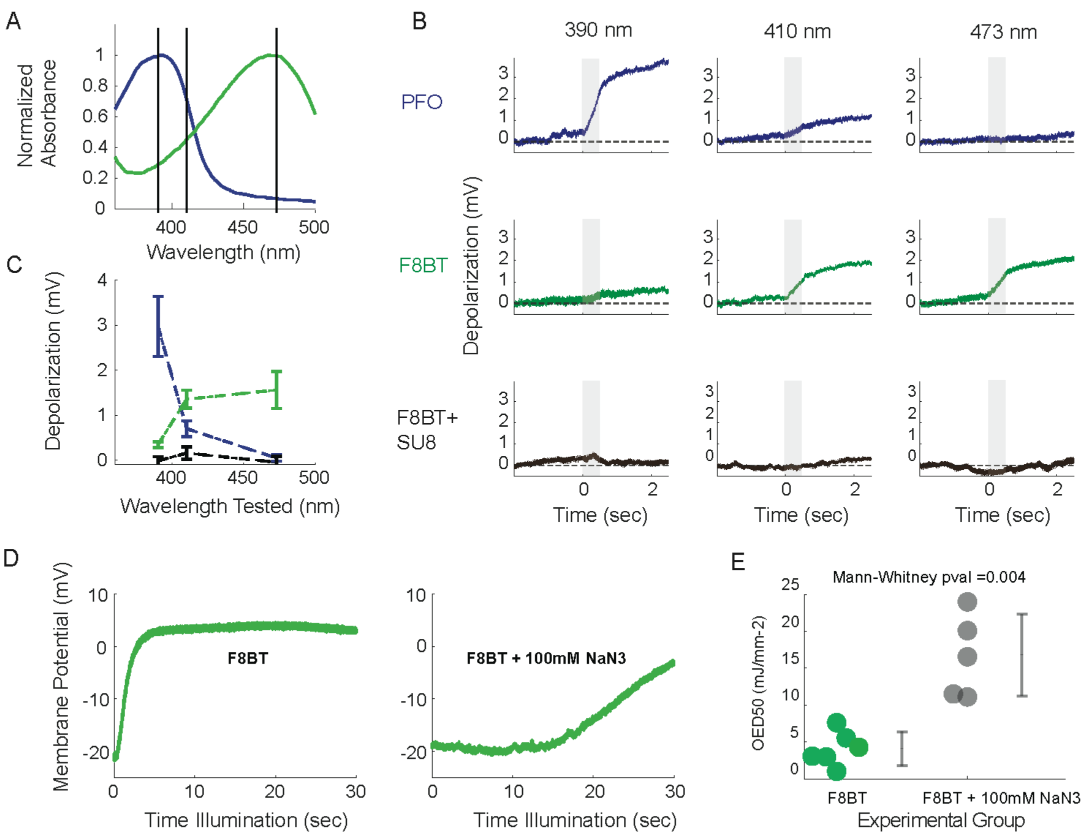

Supplementary Figure 5. Spectral dependence of irreversible depolarizations and a role for oxygen. (a) Absorbance plots of PFO and F8BT, with black lines indicating the 3 wavelengths employed for testing spectral dependence. (b) Mean trace per condition tested, rows by material, column by used irradiance wavelength. Constant power of 2.3 mW/mm^2 was used across wavelengths, 500 ms stimulus starting at t=0. (c) Quantification of spectral dependence experiment. Coating with SU8 abolishes response. (d) Testing the effect of a singlet oxygen scavenger on the depolarization rate. The figure shows a representative trace obtained from the first 30 s of illumination for a recording in standard extracellular medium (left) or in extracellular medium containing 100 mM NaN_3 under 0.86mW/mm^2 irradiation (right). (e) Quantification of the OED50 obtained per experimental condition across tested cells, showing increased IED50 with recordings in 100 mM NaN_3.
